# When Is Open Access Not Open Access?

**DOI:** 10.1371/journal.pbio.0050285

**Published:** 2007-10-16

**Authors:** Catriona J MacCallum

## Abstract

As open access grows in prominence, so too has confusion about what open access means; such confusion arises from a genuine misunderstanding of open access by funders, authors, editors, and publishers alike.

Since 2003, when *PLoS Biology* was launched, there has been a spectacular growth in “open-access” journals. The Directory of Open Access Journals (http://www.doaj.org/), hosted by Lund University Libraries, lists 2,816 open-access journals as this article goes to press (and probably more by the time you read this). Authors also have various “open-access” options within existing subscription journals offered by traditional publishers (e.g., Blackwell, Springer, Oxford University Press, and many others). In return for a fee to the publisher, an author's individual article is made freely available and (sometimes) deposited in PubMed Central (PMC). But, as open access grows in prominence, so too has confusion about what open access means, particularly with regard to unrestricted use of content—which true open access allows. This confusion is being promulgated by journal publishers at the expense of authors and funding agencies wanting to support open access.

Research funding agencies have been instrumental in driving the change toward open-access publishing. Many agencies now require the researchers they fund to make their articles freely available on publication, or within 6 or 12 months of publication (the most up-to-date information about such developments is available from the blog of Peter Suber, a leading scholar in the open-access movement [1]). The Wellcome trust, which has led the charge among funders, was also behind the recent launch of the European equivalent of PubMed Central (UKPMC; http://ukpmc.ac.uk/), a free digital archive of biomedical and life sciences journal literature, which aims to mirror that created and hosted by the National Institutes of Health in the United States (http://www.pubmedcentral.nih.gov/). European funding agencies are already responding to this resource. For example, within the United Kingdom, the Medical Research Council, the Department of Health, the British Heart Foundation, and Cancer Research UK all now require their grant holders to make their research articles available in UKPMC as soon as possible (and no later than six months) after publication. (Regular updates on open-access mandates and policies are available at http://www.eprints.org/openaccess/policysignup/).

All these initiatives signal the ongoing transition from subscription-based to open-access publishing of the scholarly research. It seems we are finally witnessing a sea change in scientific communication. But with this welcome trend comes a more insidious one to obscure the true meaning of open access by confusing it with free access. As the original Bethesda definition makes clear [2] (Box 1), open access allows for unrestricted derivative use; free access does not. So the beauty of open-access publishing is not just that you can download and read an article for personal use. You can also redistribute it, make derivative copies of it (such as reproducing it in another language; several *PLoS Biology* articles have been reproduced, in whole or in part, in Greek on http://www.biology4u.gr), use it for educational purposes (e.g., [3]), or, most importantly, for purposes that we can't yet envisage. This is because the open-access license most commonly used—the Creative Commons Attribution license (http://creativecommons.org/licenses/)—permits derivative reuse, as long as the author is correctly cited and attributed for the work. It is the most liberal of the available Creative Commons licenses (there are six), which are now applied widely to books, music, videos, etc., as well as scholarly works. It is important to note that of the six different Creative Commons licenses, only those that permit unrestricted derivative use (which may be limited to noncommercial use) truly equate with open access.

**Box 1. The Bethesda Statement on Open-Access Publishing**

This is taken from http://www.earlham.edu/~peters/fos/bethesda.htm.

An Open Access Publication^1^ is one that meets the following two conditions:

1. The author(s) and copyright holder(s) grant(s) to all users a free, irrevocable, worldwide, perpetual right of access to, and a license to copy, use, distribute, transmit and display the work publicly and to make and distribute derivative works, in any digital medium for any responsible purpose, subject to proper attribution of authorship^2^, as well as the right to make small numbers of printed copies for their personal use.
2. A complete version of the work and all supplemental materials, including a copy of the permission as stated above, in a suitable standard electronic format is deposited immediately upon initial publication in at least one online repository that is supported by an academic institution, scholarly society, government agency, or other well-established organization that seeks to enable open access, unrestricted distribution, interoperability, and long-term archiving (for the biomedical sciences, PubMed Central is such a repository).

^1^Open access is a property of individual works, not necessarily journals or publishers.

^2^Community standards, rather than copyright law, will continue to provide the mechanism for enforcement of proper attribution and responsible use of the published work, as they do now.

New York Times science writer Carl Zimmer demonstrated the distinction succinctly [4]. He discusses a recent case where Wiley threatened legal action after a neuroscience graduate posted some figures from one of their journal articles on her blog (despite the fact that this is already permitted under terms of “fair dealing” or “fair use”). His response was:

“Compare Shelley's experience to what I'm about to do. I'm going to—shudder—reprint a diagram from a journal. Just lift it straight out. ….And what do I now hear from PLOS? Do I hear the grinding of lawyerly knives? No. I hear the blissful silence of Open Access, a slowly-spreading trend in the journal world. PLOS makes it very clear on their web site that “everything we publish is freely available online throughout the world, for you to read, download, copy, distribute, and use (with attribution) any way you wish.” No muss, no fuss. If I want to blog about this paper right now, I can grab a relevant image right now from it. In fact, I just did.” [4]

Subsequent to an outcry within the blogoshpere (for a summary, see [5]), Wiley withdrew their threat of legal action. But the license that enabled Zimmer to extract the information he needed without worry is used by all the established open-access publishers, such as PLoS, BioMed Central, and Hindawi, as well as some traditional publishers, such as Oxford University Press (OUP). Examples of the license from some journals are given in Box 2, and each statement is explicit about how the article may be used. In all cases, copyright is held by the authors, although this is not strictly necessary; as long as there is unrestricted derivative use, the copyright could be held by the author or the publisher and still be regarded as open access.

Other journals purporting to be “open access” or publishers with an “open-access option” are not all that they seem. Take, for example, the journal *Molecular Systems Biology*. This is listed as an open-access journal by the DOAJ and published by the Nature Publishing Group. On the journal website (http://www.nature.com/msb/index.html), there is a prominent link in the left hand column titled “open access.” Here, you are informed that the journal “makes primary research freely available to all researchers worldwide, ensuring maximum dissemination of content through the nature.com platform.” The publisher charges a publication fee (like PLoS) and publishes their content under a Creative Commons license (also like PLoS). But that's where the similarities end. The Creative Commons license used is actually very different, despite the fact that at the bottom of the HTML version of any of their articles, there is a statement that the article is licensed under the “Attribution” license. However, when you click through to the full version, you are presented with the most restrictive Creative Commons license available, the “Attribution-Noncommercial-No Derivative Works” license. The article can still be downloaded and redistributed (for personal use), but permission from the publisher is required for any additional derivative use (see also Box 2). What exactly have the authors—or, more likely, their funding agencies—agreed to pay for here? It is certainly not open access as defined by the Bethesda Statement [2].

**Box 2. Examples of License Agreements and Open Access**

Four of the five journals, whose license statements are listed below, use a Creative Commons license (http://creativecommons.org/about/licenses/meet-the-licenses). Of these, *PLoS Biology, BMC Biology*, and *Nucleic Acids Research* conform to open access, as defined by the Bethesda Statement (Box 1). *PLoS Biology* and *BMC Biology* apply the Creative Commons “Attribution” (by) license to their articles, whereas *Nucleic Acids Research* applies the Creative Commons “Attribution Non-commercial” (by-nc) license.

*Molecular Systems Biology* also applies a Creative Commons license to their articles, but this journal provides free access rather than open access, because they do not allow any derivative works to be made without permission. The particular license they use is the Creative Commons “Attribution Non-commercial No derivatives” (by-nc-nd) license.

The journal *Proceedings of the National Academy of Sciences* (*PNAS*) does not use a Creative Commons license, but they do have a free access option. Their articles can be used for a wide range of purposes without permission, but the creation of derivative works is still restricted so this option is also not open access.

**Open Access (Permits Unrestricted Derivative Use):**

- *PLoS Biology* (http://plosbiology.org)
**Copyright:** © 2007 Jetz et al. This is an open-access article distributed under the terms of the Creative Commons Attribution License, which **permits unrestricted use**, distribution, and reproduction in any medium, provided the original author and source are credited.
- *BMC Biology* (http://www.biomedcentral.com/bmcbiol/)
  © 2007 Hoffman and Goodisman; licensee BioMed Central Ltd.
  This is an Open Access article distributed under the terms of the Creative Commons Attribution License (http://creativecommons.org/licenses/by/2.0), **which permits unrestricted use**, distribution, and reproduction in any medium, provided the original work is properly cited.
- *Nucleic Acids Research* (http://nar.oxfordjournals.org/) © 2007 The Author(s)
  This is an Open Access article distributed under the terms of the Creative Commons Attribution Non-Commercial License (http://creativecommons.org/licenses/by-nc/2.0/uk/) **which permits unrestricted non-commercial use**, distribution, and reproduction in any medium, provided the original work is properly cited.

**Free Access (Does Not Permit Unrestricted Derivative Use):**

- *Molecular Systems Biology* (http://www.nature.com/msb/index.html)
  This is an open-access article distributed under the terms of the Creative Commons Attribution License, which permits distribution, and reproduction in any medium, provided the original author and source are credited. **This license does not permit commercial exploitation or the creation of derivative works without specific permission.**
  © 2007 EMBO and Nature Publishing Group All rights reserved (on the pdf).
- *PNAS* (http://www.pnas.org/)
  Freely available online through the PNAS open access option.
  © 2007 by The National Academy of Sciences of the USA.

Some journals do not claim to be fully open access but provide an “open-access option” that permits articles to be deposited in PMC and thus conforms to the minimum guidelines set by, e.g., the Wellcome Trust. But confusion abounds here as well. If you search various journal sites for “open-access” options, you'll find it enormously difficult to obtain clearly labeled information describing how you can use the article. Often an article is free to read, and has been deposited in the US PMC, but it's not clear from the terms on the article that it can be distributed freely to others or reused without explicit permission from the appropriate permissions department. Again, this is not open access.

A particularly befuddling example comes from the *Journal of Biological Chemistry* (JBC). The JBC website states that it is an “open-access” journal and that their publisher—the American Society for Biochemistry and Molecular Biology (ASBMB)—is an “open-access” publisher (http://www.jbc.org/misc/JBC_Open_Access.shtml). They don't charge a publication fee as such but do have page charges like many traditional journals (US$75 per published page). What you actually find is that manuscripts that are currently in press are available for free but those published in the current issue are not. On the table of contents of the current issue, there is a helpful permissions button next to each article that links to a webpage that helps you calculate the charges for different uses (even for posting on the internet). It is unclear how this qualifies as open access.

Does the distinction between free and open access really matter if anyone can read the article for free? Isn't open access just about making the literature available? Well, yes and no. Free access is certainly important, but it's only the starting point. At least of equal importance is the potential for innovation. We don't know yet what innovation means with regards to the full text of an article—who could have predicted the impact GenBank would have or the uses that sequences are now being put to? As one colleague put it, free access is like giving a child a Lego car and telling her that she can look at it, perhaps touch it, but certainly not take it apart and make an airplane from it. The full potential of the work cannot be realized [6].

What's worrying is that there are already examples of publishers restricting use of their “free-access” articles, even in international repositories. For example, some of the publishers that currently allow their articles to be deposited in the US PMC will not allow those same articles to be mirrored and made available from the UK site (a list of these journals can be found at http://ukpmc.ac.uk/ppmc-localhtml/not_in_ukpmc.html). It's hard to understand the reasoning for this limitation—after all, the articles are freely available from the US site. But what's disturbing is that publishers can act like this because the articles themselves are not truly open access—who knows what further restrictions might be placed on these articles in the future.

So although true open access is unquestionably good for science, there is a real concern that the precise meaning of the term is being corrupted and eroded. Not all of the confusion about open access that currently permeates the scholarly publishing industry is likely to be intentional (at least not all of it); much arises from a genuine misunderstanding of open access by funders, authors, editors, and publishers alike. However, no matter how unintentional such obfuscation might be, it is detrimental to the free exchange and use of scholarly research. It is now time for all publishers to tighten the definition and application of open access and be clearer about the uses and restrictions applied to their articles. Open access is a term that should only be used when the license permits both free access and unrestricted derivative use (and gives appropriate attribution). Authors and funders need to be much more aware of the small print before inadvertently signing away their rights and those of their readers and, even worse, paying good money for the privilege.

Perhaps the real key to establishing a broad consensus around the meaning of open access will be the development of resources that demonstrate the potential of unrestricted reuse of the literature**—**the “Lego factor.” If certain work is not included in these resources because of restrictive license agreements, authors will probably pay much closer attention to the claim that a publisher is “open access.” Enlightened self-interest can be a powerful force.

## References

[pbio-0050285-b001] Suber P Open access news. http://www.earlham.edu/~peters/fos/fosblog.html.

[pbio-0050285-b002] (2003). http://www.earlham.edu/~peters/fos/bethesda.htm.

[pbio-0050285-b003] Millman SP, Anderson B (2004). Monkey see, monkey do: Comparing baboon and human development.

[pbio-0050285-b004] Zimmer C (2007). An open mouse. Scitizen. http://www.scitizen.com/screens/blogPage/viewBlog/sw_viewBlog.php?idTheme=31&idContribution=652.

[pbio-0050285-b005] Coturnix (26 April 2007 12:53). Fair use and open science. A blog around the clock. http://scienceblogs.com/clock/2007/04/fair_use_and_open_science.php.

[pbio-0050285-b006] Surridge C (7 March 2007 05:13). Free but not open?. PLoS Blogs.

